# The therapeutic and prognostic role of cuproptosis-related genes in triple negative breast cancer

**DOI:** 10.1186/s12859-023-05348-3

**Published:** 2023-05-31

**Authors:** Bingye Shi, Wei Zhang, Tao Wang, Zhenyu Cui

**Affiliations:** 1 Color Ultrasound Room, Affiliated Hospital of Hebei University, Baoding, Hebei China; 2 Medical Engineering Center, Affiliated Hospital of Hebei University, Baoding, Hebei China; 3 Department of Integrated Traditional Chinese and Western Medicine, Affiliated Hospital of Hebei University, Baoding, Hebei China; 4 Department of Urology Surgery, Affiliated Hospital of Hebei University, Baoding, Hebei China

**Keywords:** Triple negative breast cancer, Cuproptosis, Bioinformatics, Immune infiltration, Survival, Drug sensitivity

## Abstract

**Background:**

This study aimed to observe the potential impact of known cuproptosis-related genes (CRGs) on triple negative breast cancer (TNBC) development, as well as their associated molecular mechanisms, immune infiltration mechanisms and potential therapeutic agents.

**Results:**

Based on the Cox Proportional Hazard Model, 11 CRGs may be especially important in TNBC development and progression (considered as the Key-TNBC-CRGs). The expression of several Key-TNBC-CRGs (e.g., *ATP7A, PIK3CA, LIAS,* and *LIPT*) are associated with common mutations. The SCNA variation of 11 Key-TNBC-CRGs are related to differences immune infiltration profiles. In particular, depletion of *ATP7A*, *ATP7B*, *CLS*, *LIAS*, and *SCL31A1* and while high amplification of *NLRP3* and *LIPT2* are correlated with decreased immune infiltration. In our Cox proportional hazards regression model, there is a significant difference in the overall survival between high-risk and low-risk groups. The HR in the high-risk group is 3.891 versus the low-risk group. And this model has a satisfactory performance in Prediction of 5–15-year survival, in particular in the 10-year survival (AUC = 0.836). Finally, we discovered some potential drugs for TNBC treatment based on the strategy of targeting 11 Key-TNBC-CRGs, such as Dasatinib combined with ABT-737, Erastin or Methotrexate, and Docetaxel/Ispinesib combination.

**Conclusion:**

In conclusion, CRGs may play important roles in TNBC development, and they can impact tumor immune microenvironment and patient survival. The Key-TNBC-CRGs interact mutually and can be influenced by common BC-related mutations. Additionally, we established a 11-gene risk model with a robust performance in prediction of 5–15-year survival. As well, some new drugs are proposed potentially effective in TNBC based on the CRG strategy.

## Background

Triple negative breast cancer (TNBC, lacking the expression of ER, PR, and HER2) is an extremely aggressive form of breast cancer (BC). TNBC has a high risk of metastasis, a poor prognosis and lacks the efficacy of conventional targeted therapies in other BC subtypes [1]. Once metastasis has occurred, the median survival is short. Commonly, TNBC generally considered to be the most malignant subtype of BC [2, 3], and it is therefore of particular importance to investigate the mechanisms of its development and progression. The vast majority of TNBCs are categorized as overlapping with the basal-like breast cancer (BLBC) subtype, which is characterized by the expression of basal/myoepithelial signatures and mesenchymal markers suggesting the occurrence of EMT.

Copper homeostasis is essential for various enzymatic reactions and the organ-function metabolic processes; also, copper excess and aggregation can cause oxidative stress and cytotoxicity [4]. Cuproptosis is a newly discovered form of cell death that depends on mitochondrial respiration; it is distinct from other known cell-death mechanisms (e.g., apoptosis, ferroptosis, pyroptosis, and necroptosis) [5]. It is a kind of non-apoptotic programmed cell death induced by the accumulation of intracellular copper [6]. The best-known mechanism of cuprotosis is targeting lipoylated TCA cycle proteins, especially regulated by mitochondrial ferredoxin 1-mediated protein lipoylation [5]. Copper accumulates in the cells and directly binds to lipoylated components of the tricarboxylic acid cycle. This leads to abnormal aggregation of the lipoylated protein and the subsequent loss of the iron-sulfur cluster protein, which together result in proteotoxic stress and ultimately cell death [7].

Recently, very few studies have reported the association between cuproptosis and BC progression (as well as the treatment outcomes, immunogenicity, and immunotherapy responses). In other oncology fields, there are also not many related studies. In bladder cancer, the cuproptosis scoring system has been developed to predict the clinical outcome and immune response [8]. The development and progression of bladder cancer are likely to be influenced by cuproptosis, which may involve a diverse and complex tumor microenvironment (TME) [8]. Similarly, the molecular subtypes of lung adenocarcinoma based on cuproptosis-related genes can be used as a promising biomarker, which is of great importance to distinguish the relationship between cuproptosis and the immune microenvironment [9]. So far, cuproptosis-related modification patterns have been reported to depict the tumor microenvironment/immunotherapy/prognosis in kidney renal clear cell carcinoma [10], clear cell renal cell carcinoma [11], hepatocellular carcinoma [12], and colorectal cancer [13]. Among the limited bioinformatics studies in BC, it is true that estimating cuproptosis patterns in tumors could help predict the prognosis and characteristics of TME cell infiltration and guide more effective chemotherapeutic and immunotherapeutic strategies [7]. However, current BC studies based on publicly available data only use the overall BC cases, and in particular, there are very limited research of cuproptosis involvement in the prognosis of TNBC by bioinformatic analysis [14, 15]. Given that TNBC is the most malignant subtype of BC, it is particularly significant to explore the potential mechanisms of progression and treatment strategies. Cuproptosis may be involved in the development and progression of TNBC in all possibility. This study aimed to observe the potential impact of known cuproptosis-related genes (CRGs) on the survival of TNBC patients through bioinformatics analysis, as well as to explore their associated molecular mechanisms, immune infiltration mechanisms and potential therapeutic agents.

## Results

### TNBC associated CRGs

**Fig. 1:**
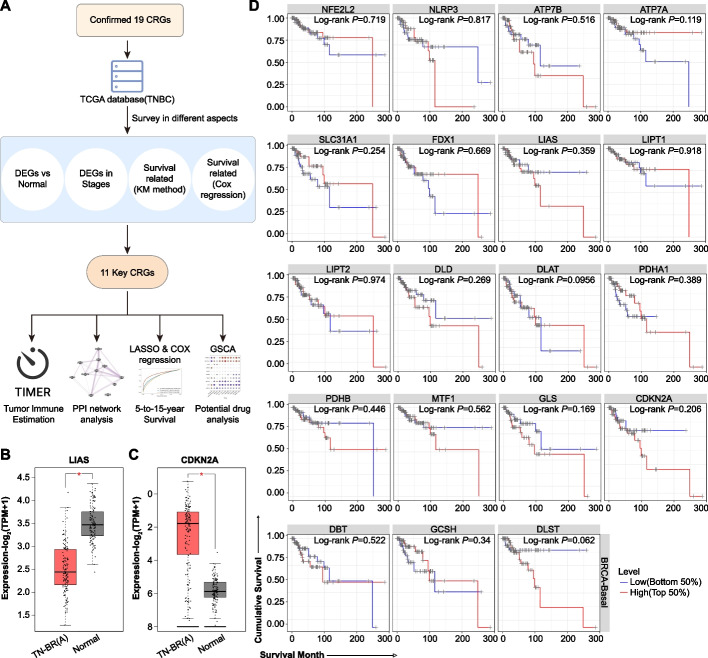
The flowchart of this bioinformatics analysis and differential expression of 19 CRGs and relationship between survival and CRGs. **A** The flowchart of this study. **B**–**C** In comparison with the normal tissue, only **B** LIASwas significantly decreased and **C** CDKN2A was significantly increased. **D** In the Kaplan–Meier method, no differences in the overall survival are discovered between the high and low groups for each CRG

First, the expression of 19 CRGs were analyzed based on RNA-sequencing raw data of The Cancer Genome Atlas (TCGA) database. In comparison with the normal tissue, only LIAS was significantly decreased and CDKN2A was significantly increased (Fig. 1A, B). Besides, not any CRG was associated with the major stage progression (*P* > 0.05 for each CRG). Next, using the Kaplan–Meier method, the relationship between survival and CRGs was probed, but we discovered not any significant difference in the overall survival between the high and low groups for each CRG (Fig. 1C). Next, we applied the Cox Proportional Hazard Model, in combination with Purity, Major stage and Age. The features in the Cox Proportional Hazard Model were presented in Table 1. The overall performance of the Cox Proportional Hazard Model was as following: Rsquare = 0.3; Likelihood ratio test *p* = 5.84e−03; Wald test *p* = 3.27e−305; and Log rank test *p* = 2.04e−03. Next, we screened all the CRGS with a *p* value less than 0.1. These genes may be especially important in TNBC development and progression (Fig. 1D). Together, there were 11 CRGs: *NFE2L2, NLRP3, ATP7B, ATP7A, SLC31A1, LIAS, LIPT1, DLAT, PDHB, GLS* and *DLST*. These 11 CRGs were considered as the Key-TNBC-CRGs.

**Table 1:** The risks of CRGs in the cox proportional hazard model of TNBC

Features	Coef	HR	HR 95% CI		*p* value	Significance
Purity	4.262	70.973	8.718	5.778090e+02	0.000	*** (*p* ≤ 0.001)
Stage2	11.221	74,712.949	25,590.291	2.181306e+05	0.000	***
Stage3	14.346	1,699,753.178	592,717.2	4.874434e+06	0.000	***
Stage4	17.631	45,386,564.737	4,067,480.4	5.064413e+08	0.000	***
Age	0.022	1.023	0.984	1.062000	0.250	
*NFE2L2*	− 1.823	0.162	0.073	0.358	0.000	***
*NLRP3*	2.379	10.797	5.438	21.437	0.000	***
*ATP7B*	1.547	4.698	2.236	9.871	0.000	***
*ATP7A*	− 1.142	0.319	0.151	0.673	0.003	** (0.05 < p < 0.001)
*SLC31A1*	− 1.185	0.306	0.141	0.662	0.003	**
*FDX1*	0.318	1.374	0.622	3.037	0.432	
*LIAS*	0.787	2.197	0.941	5.125	0.069	·
*LIPT1*	0.402	1.495	0.499	4.486	0.473	
*LIPT2*	0.923	2.516	0.886	7.144	0.083	·
*DLD*	0.234	1.264	0.634	2.52	0.506	
*DLAT*	− 1.180	0.307	0.164	0.577	0.000	***
*PDHA1*	0.144	1.155	0.513	2.602	0.728	
*PDHB*	1.533	4.630	1.285	1.669	0.019	* (p ≤ 0.05)
*MTF1*	0.273	1.313	0.581	2.97	0.512	
*GLS*	0.530	1.699	1.067	2.705	0.026	*
*CDKN2A*	− 0.045	0.956	0.772	1.182	0.676	
*DBT*	0.596	1.815	0.578	5.695	0.307	
*GCSH*	− 0.516	0.597	0.310	1.148	0.122	
*DLST*	2.625	13.804	4.360	43.704	0.000	***

*, **, *** respectively represent p values less than or equal to 0.05, p values between 0.05 and 0.001, and p values less than or equal to 0.001 after two-tailed T-test calculation

### Relationship between common mutations in BC and the expression of Key-TNBC-CRGs

**Fig. 2:**
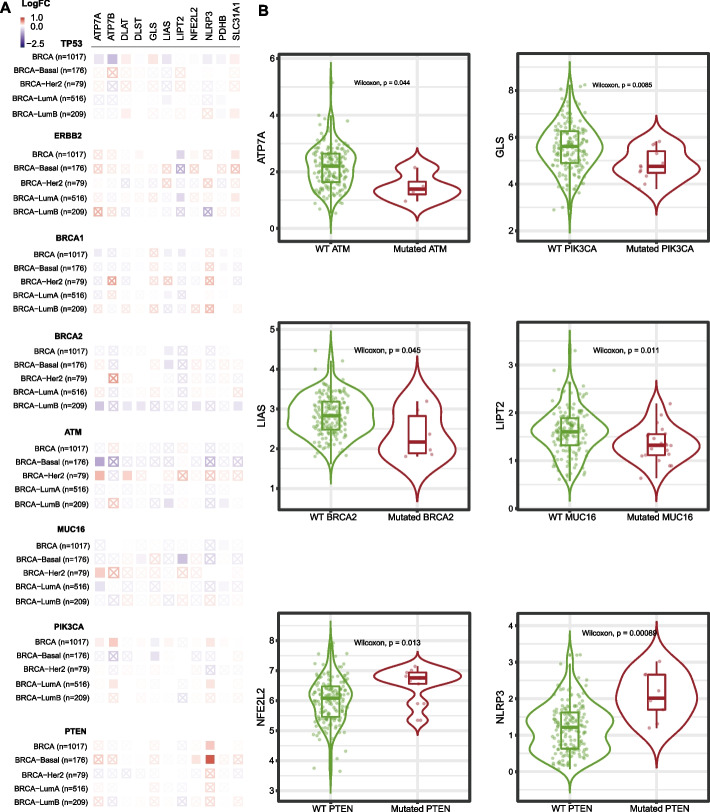
Relationship between common mutations in BC and the expression of Key-TNBC-CRGs. **A** Common mutations in BC (including TP53, ERBB2, BRCA1, BRCA2, ATM, MUC16, PIK3CA, and PTEN) have potential influence in the expression of 11 Key-TNBC-CRGs. **B** The Violin diagrams of significantly changed expression of CRGs in mutation versus WT. ATP7A is decreased in the ATM mutant group; PIK3CA is decreased in the GLC mutant group; LIAS is decreased in the BRCA2 mutant group; LIPT is decreased in the MUC16 mutant group. Meanwhile, the expression of NFE2L2 and NLRP3 are increased in the PTEN mutant group

Common mutations in BC (including TP53, ERBB2, BRCA1, BRCA2, ATM, MUC16, PIK3CA, and PTEN) were analyzed to probe the potential influence in the expression of Key-TNBC-CRGs through the TIMER tool. The changes in 11 key TNBC-CRGs associated with the above mutations are shown in Fig. 2 (Fig. 2A: the overall statistical relevance; Fig. 2B: the violin plots of significantly altered expression of CRGs in mutation vs. WT). ATP7A is decreased in the ATM mutant group; PIK3CA is decreased in the GLC mutant group; LIAS is decreased in the BRCA2 mutant group; LIPT is decreased in the MUC16 mutant group. Meanwhile, the expression of NFE2L2 and NLRP3 are increased in the PTEN mutant group.

### Immune infiltration levels associated with Key-TNBC-CRGs

**Fig. 3:**
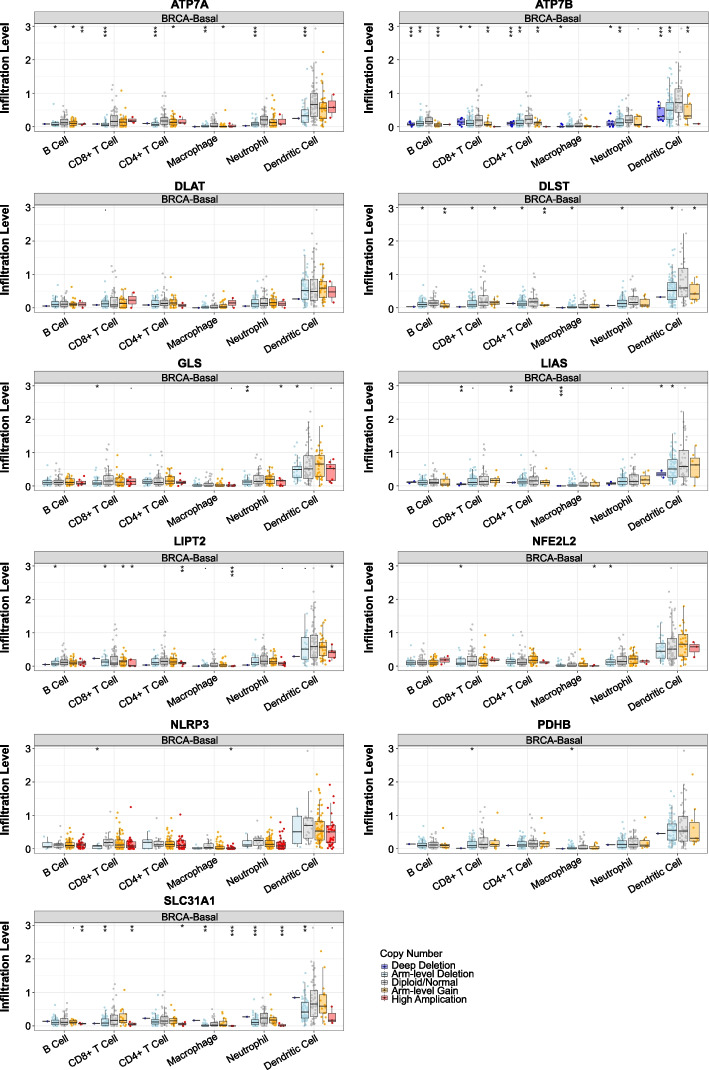
Immune infiltration levels associated with Key-TNBC-CRGs. The somatic copy number alterations (SCNA) variation of 11 Key-TNBC-CRGs are related to different immune infiltration profiles. In particular, depletion of ATP7A, ATP7B, CLS, LIAS, and SCL31A1 are correlated with decreased immune infiltration; while high amplification of NLRP3 and LIPT2 are related to decreased immune infiltration

According to the SCNA module of the TIMER tool, the SCNA variation of 11 Key-TNBC-CRGs are related to different immune infiltration profiles (Fig. 3). In particular, depletion of ATP7A ATP7B, CLS, LIAS, and SCL31A1 are correlated with decreased immune infiltration; while high amplification of NLRP3 and LIPT2 are related to decreased immune infiltration.

### CRG interaction networks

**Fig. 4:**
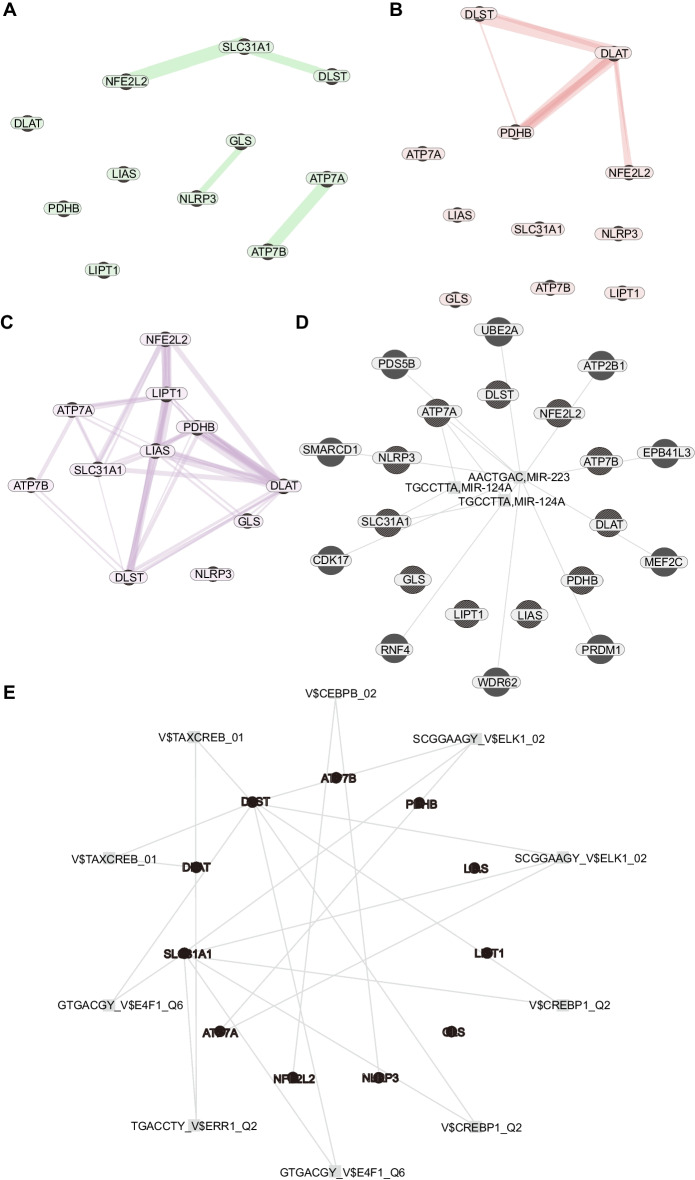
The interaction networks of 11 Key-TNBC-CRGs. **A** The Genetic interaction network, *NFE2L2*, *SLC31A1*, *DLST*, *NLRP3*, *GLS*, *ATP7B*, and *ATP7A* may be the hub genes. **B** The protein physical interaction, DLST, PDHB, DLST and NFE2L2 are connected with a network. **C** The Co-expression networks. NFE2L2, LIPT1, SLC31A1, DLST, NLRP3, GLS, ATP7B, DLAT, and ATP7A are mutually interacted. **D** The linked miRNA networks, in which miR-124A and miR-223 may play important roles in the interaction of the 11 Key-TNBC-CRGs. **E** The transcriptional-factor prediction network, these CRGs may be regulated by CREB, ELK1, E4F1, ERR1, and CEBPB

The CRG interaction networks are shown in Fig. 4. In the Genetic interaction network, *NFE2L2, SLC31A1, DLST, NLRP3, GLS, ATP7B*, and *ATP7A* may be the hub genes (Fig. 4A). In protein physical interaction, DLST, PDHB, DLST and NFE2L2 are connected with a network (Fig. 4B). In the Co-expression network, NFE2L2, LIPT1, SLC31A1, DLST, NLRP3, GLS, ATP7B, DLAT, and ATP7A are mutually interacted (Fig. 4C). In the linked miRNA network, miR-124A and miR-223 may play important roles in the interaction of the 11 Key TNBC CRGs (Fig. 4D). In the transcriptional-factor prediction network (Fig. 4E), these CRGs may be regulated by CREB, ELK1, E4F1, ERR1, CEBPB, etc., which may be potential targets in TNBC treatment using the CRG-related strategy.

### Prediction of 5–15-year survival by the Cox regression model

In the generated Cox proportional hazards regression model, the equation of the risk score is as follow. Riskscore = (− 0.7315) *NFE2L2 + (0.3389) *NLRP3 + (0.752) *ATP7B + (− 0.6176) *ATP7A + (-0.0986) *SLC31A1 + (0.4263) *LIAS + (0.4111) *LIPT1 + (− 0.1973) *DLAT + (0.2469) *PDHB + (0.3716) *GLS + (0.9804) *DLST.

**Fig. 5:**
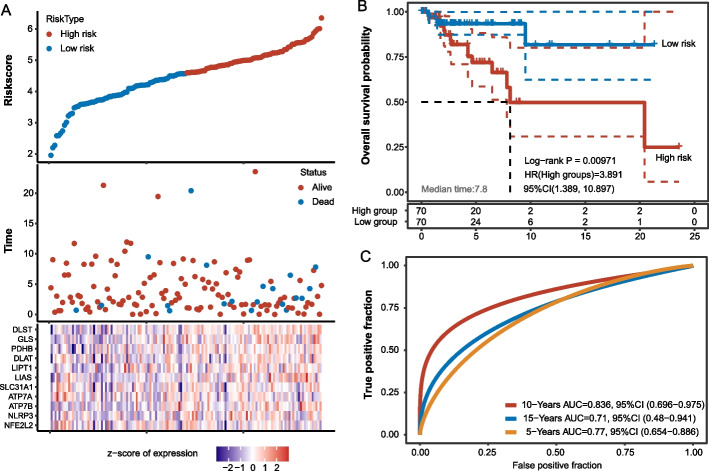
Prediction of 5–15-year survival by the Cox regression model. **A** The risk scores and the survival time with corresponding level of CRGS in TNBC cases from the TCGA database. **B** The median survival time is 7.8 months, and there is a significant difference in the overall survival between high-risk and low risk groups. The HR in the high-risk group is 3.891 versus the low-risk group. **C** The ROC curve of the cox regression model in prediction of 5–15-year survival

The risk scores and the survival time with corresponding level of CRGS in all TCGA cases are shown in Fig. 5A. The median survival time is 7.8 months, and there is a significant difference in the overall survival between high-risk and low-risk groups (Fig. 5B, Log-rank *p* =  < 0.01). The HR in the high-risk group is 3.891 versus the low-risk group. And this model has a satisfactory performance in Prediction of 5–15-year survival, in particular in the 10-year survival (AUC = 0.836) (Fig. 5C).

### Potential drugs for TNBC based on Key TNBC CRGs

**Fig. 6:**
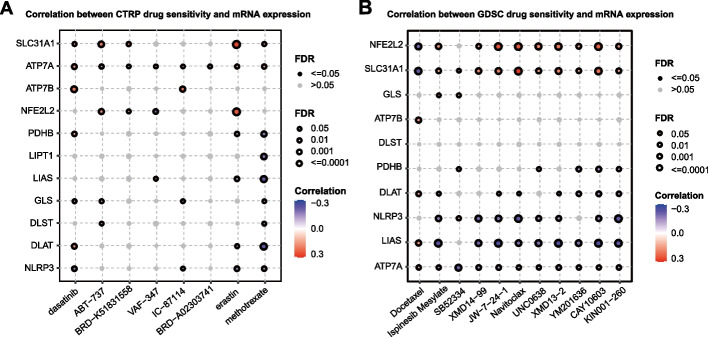
Potential drugs for TNBC based on Key-TNBC-CRGs. **A** The analysis of drug sensitivity based on the CTRP database. Dasatinib maybe effective, especially when combined with ABT-737, Erastin and Methotrexate. **B** The analysis of drug sensitivity based on the GDSC database. Docetaxel and Ispinesib combination may be efficacy in TNBC treatment

Finally, using the CTRP and GDSC databases, we discovered some potential drugs for TNBC treatment based on the strategy of targeting 11 key TNBC CRGs. As shown in Fig. 6A, according to the analysis of CTRP database, dasatinib maybe effective, especially when combined with ABT-737, erastin and methotrexate. According to the GDSC database analysis (Fig. 6B), Docetaxel and Ispinesib combination may be efficacy in TNBC treatment. The future work can evaluate the performance of above drugs (or drug combination) in vitro or in vivo.

## Discussion

It is known that disturbed copper homeostasis may influence cancer progression, through different mechanisms [16, 17]. The novelty and technicality of this study lie in that: the role of CRGs on the development, survival, and immune infiltration of TNBC is not fully clear; sensitization strategies based CRGs have not been proposed; furthermore, which CRGs are the most critical in TNBC and whether key CRGs can be used to construct a prognostic model are addressed for the first time in this study. To date, the limited studies about cuproptosis and BC are as follow. Recently, a study used a cuproptosis-related gene signature (*PGK1, SLC52A2, SEC14L2, RAD23B, SLC16A6, CCL5*, and *MAL2*) and constructed a scoring system to quantify the cuproptosis pattern of BRCA patients [18]. In that work, patients in the low-CRG score group were characterized by higher immune cell infiltration, immune checkpoint expression, immune checkpoint inhibitor scores, and greater sensitivity to immunotherapy; and they found that RAD23B was a favorable target associated with BC progression, drug resistance, and poor prognosis. In another article published recently, expression of FDX1, DLD, DLAT, LIAS, LIPT1, GLS MTF1, and PDHA1 was downregulated, while CDKN2A expression level was elevated in BC [19]; poor survival was associated with high levels of CDKN2A and PDHA1 and low levels of MTF1, DLD, LIPT1, and FDX1. Moreover, they constructed a cuproptosis-related signature with six genes (*DKN2A, MTF1, PDHA1, DLD, LIPT1,* and *FDX1*) for in OS prediction. Also in 2022, a similar study created a prognostic profile using 19 CRGs and 37 cuproptosis-related lncRNAs (with AUC = 0.766, 0.808, and 0.745 for 1-year, 2-year, and 3-year survival) [20]. Another work in the same year identified a set of 11 cuproptosis-related lncRNAs, based on which to construct the risk model. The AUC values for ROC of 1-, 3-, and 5-year risk were 0.849, 0.779, and 0.794, respectively [21].

In these studies, positive results were easily obtained due to the relatively large sample size (for overall BC cases); in contrast, we used TNBC samples, with a relatively small size, and the results were not consistent with the overall BC population. For example, there are only 2 DEGs (TNBC vs. Control) in our 19 candidate CRGs, and not any Key-TNBC-CRG is survival-correlated gene singly. It is also evident that TNBC and other BCs are significantly heterogeneous, and TNBC is more difficult to target.

In the Cox regression model, we discovered 11 key CRGs: *NFE2L2, NLRP3, ATP7B, ATP7A, SLC31A1, LIAS, LIPT1, DLAT, PDHB, GLS* and *DLST*. Currently, there are few studies on these genes in TNBC. Some of the important findings are as follows. NFE2L2 was identified as one of top 10 targets of Curcumin against TNBC [22]. NLRP3 may mediate the cisplatin induced pyroptosis via activation in TNBC (via activation of MEG3/NLRP3/caspase-1/GSDMD pathway) [23]. Similarly, Berberine can affect tumor outgrowth and spontaneous metastasis in TNBC through a mechanism associated with inhibition the NLRP3 inflammasome pathway [24]. Glutaminase (GLS), a key enzyme for glutamine metabolism, can improve antitumor T cell activation in both a spontaneous mouse TNBC model and orthotopic grafts [25]. It is regarded to be essential for the growth of TNBC cells through glutamine metabolism pathway and mTOR inhibition [26]. Moreover, dihydrolipoamide S-succinyltransferase (DLST) is an important tricarboxylic acid (TCA) cycle enzyme, it has been reported to be useful in predicting survival among TNBC patients; DLST depletion suppresses growth and induces death in subsets of human TNBC cell lines; in particular, DLST depletion in sensitive TNBC cells results in elevated ROS levels while N-acetyl-L-cysteine partially can rescue cell growth [27]. So far, none of the above mechanisms of CRG involvement in TNBC onset/progression/drug-resistance has been shown to be correlated with copper death. The present study provides a new perspective for understanding their role in TNBC.

In this work, we established a Cox regression model, and the risk score in this model is efficacy in prediction of 5-, 10-, and 15-year survival. To our knowledge, this is the first model using CRGs to predict TNBC patients over 5–15 years and it performs well. The AUC of ROC is 0.836 in the 10-year survival prediction, and this accuracy does not fall under the efficacy of CRGs in other tumors or subtypes. For example, in the total BC cases, a recent study used 13 CRGs and created a model that had an AUC of only about 0.68 in 5-year prediction [28]. The similar CRG model (after the LASSO regression) in ovarian cancer showed an AUC about 0.7 [29]. And other frontier studies showed following AUC values in CRG prognostic models: 0.898 (11 genes) in IgA nephropathy [30], 0.658 for 5-year survival in clear cell renal cell carcinoma [31], less than 0.6 for 3-year survival in lung adenocarcinoma [32], around 0.63 for 5-year survival in lung adenocarcinoma [9], and around 0.57 for 5-year survival in colon cancer [33]. Thus, the CRG strategies have not performed excellently in other cancers, whereas we found them to be of outstanding value in the prognosis of TNBC, which is urgently needed for clinical application.

Still, this work has its limitation. Currently, we have only analyzed the TCGA data but have not yet conducted validation in the real world. In the future, our prognostic model will be optimized based on the specific characteristics of Chinese population. Moreover, the potentially sensitive drugs tapped in the CTRP and GDSC database have also not been validated by in-vitro or in-vivo experiments. So far this study lacks validation set to verify all the findings. Therefore, more convincing evidence is need, especially for potential sensitization strategy (we will conduct cellular experiments for validation at a later stage). Validation experiments could help accelerate the expansion of novel regimens for the treatment of TNBC. Finally, we here mainly focused on mRNAs in TNBC. The advancement of interaction prediction research in various fields of computational biology have provide valuable insights into genetic markers and lncRNAs related with TNBC, such as miRNA-lncRNA interaction prediction [34–39]. Our further study will include the role of miRNA-lncRNA interaction in the CRG-associated mechanisms in TNBC development.

## Conclusion

In conclusion, CRGs may play an important role in the development of TNBC and may influence the tumor immune microenvironment and patient survival. The key TNBC CRGs interact with each other and may be influenced by common BC-related mutations. In addition, we have established an 11-gene risk model with robust performance in predicting 5–15-year survival. As well, some new drugs are proposed potentially effective in TNBC based on the CRG strategy.

## Methods

### Selection of cuproptosis-related genes

The definite cuproptosis-related genes (CRGs) were acquired from published articles [5, 31, 40]. After reviewing, following 19 CRGs (coding genes) were used: *NFE2L2, NLRP3, ATP7B, ATP7A, SLC31A1, FDX1, LIAS, LIPT1, LIPT2, DLD, DLAT, PDHA1, PDHB, MTF1, GLS, CDKN2A, DBT, GCSH,* and *DLST*.

### CRG Expression differences in TNBC

The expression of 19 CRGs were analyzed based on RNA-sequencing raw data of The Cancer Genome Atlas (https://portal.gdc.cancer.gov/) using the gepia2 tool. Only the BRCA-basal subtype was analyzed.

### Survival associated CRGs

Using the Tumor immune estimation resource (TIMER) online tool, the links between CRGs and survival of TNBC patents were analyzed. First, the association between each single CRG and the OS and RFS was explored. In fact, no significant association was found between the expression level and the survival of TNBC patients for any of the single CRGs. The Kaplan–Meier plots for CRGs were drawn to visualize the survival differences. Levels are divided into low and high levels, and P-value of log-rank test for comparing survival curves of two groups was presented in each plot. Next, the outputs of Cox’s regression model (by function Coxph from R package survival) were presented, with HR value showing the hazard ratio, as well as its lower and upper 95% confidential interval (CI). We incorporated the Purity, stage, and Age into the Cox Proportional Hazard Model, and the CRGs with a *p* value less than 0.1 were selected as the key CRGs in TNBC (Key-TNBC-CRGs).

### Relationship between common mutations in BC and the expression of Key-TNBC-CRGs

Again, using the Gene-Mutation module of the TIMER V2.0 tool, we compared the differential gene expression between different mutation status. The heatmap was generated to show the log2 fold changes (FC) of each CRG in comparison of mutation versus WT. Following common mutations were concerned: *TP53, ERBB2, BRCA1, BRCA2, ATM, MUC16, PIK3CA,* and *PTEN*. All BC cases were analyzed, and we focused on the BRCA-Basal set. The significantly changed expression of CRGs in mutation versus WT were shown in Violin diagrams.

### Tumor immune infiltration levels associated with Key-TNBC-CRGs

The SCNA module of the TIMER tool was applied for comparison of tumor infiltration levels among tumors with different somatic copy number alterations (SCNAs) for each Key-TNBC-CRG. SCNAs include five classes: deep deletion (− 2), arm-level deletion (− 1), diploid/normal (0), arm-level gain (1), and high amplification (2). Box plots were presented to show the distributions of each immune subset at each copy number status. The infiltration level for each SCNA category was compared with the normal using a two-sided Wilcoxon rank-sum test.

### CRG interaction networks

Using the GeneMANIA online tool, we explored the connections among CRGs. In particular, following interactions were probed based on the 11 Key-TNBC-CRGs: Genetic interaction (Two genes are functionally associated if the effects of perturbing one gene were found to be modified by perturbations to a second gene. These data are collected from primary studies and BioGRID); protein physical interaction (Two gene products are linked if they were found to interact in a protein–protein interaction study. These data are collected from primary studies found in protein interaction databases, including BioGRID and PathwayCommons); Co-expression (Two genes are linked if their expression levels are similar across conditions in a gene expression study. Data are collected from the GEO database); linked miRNAs (the common linked miRNAs with two CRGs); transcriptional-factor prediction (Transcription factor targets from MSigDB).

### Prediction of 5–15-year survival by the Cox regression model

We evaluated different available models for survival prediction, including LASSO regression and multifactorial cox regression using the 11 Key-TNBC-CRGs. The LASSO regression algorithm was used for feature selection (tenfold cross-validation), and the R package glmnet was used for the analysis. And Multivariate cox regression analysis was used to construct a prognostic model (using the R package survival). RNA-sequencing expression profiles and corresponding clinical information for TNBC were downloaded from the TCGA dataset (https://portal.gdc.com). The counts data were converted to TPM and log2 (TPM + 1) was calculated; Log-rank test was used to compare differences in survival between these groups. In the Kaplan–Meier curves, the p-values and hazard ratio (HR) with 95% confidence interval (CI) were generated through log-rank tests, and univariate cox proportional hazards regression.

### Potential drugs for TNBC based on Key-TNBC-CRGs

The Gene Set Cancer Analysis tool was employed to dig potential drugs for TNBC based on Key-TNBC-CRGs. Two databases were referenced to acquire the IC50 value of drugs: Genomics of drug sensitivity in cancer (GDSC) and Genomics of therapeutics response portal (GTRP). In the GDSC set, the IC50 of 265 small molecules in 860 cell lines and its corresponding mRNA gene expression were extracted; and in the CTRP set, the IC50 of 481 small molecules in 1001 cell lines and its corresponding mRNA gene expression were extracted. The mRNA expression data and drug sensitivity data were merged. Pearson correlation analysis was performed to get the correlation between gene mRNA expression and drug IC50. The *p*-value was adjusted by the FDR value. In the generated bubble plots, we summarized the correlations between key CRGs and drugs. Blue bubbles represent negative correlations, red bubbles represent positive correlations, the deeper of color, the higher of the correlation. Bubble size is positively correlated with the FDR significance. The black outline border indicates FDR ≤ 0.05. The drugs are ranked by the integrated level of correlation coefficient and FDR of CRGs, and only the top 30 ranked drugs were presented.

## Data Availability

All data generated or analyzed in this study were sourced from the Cancer Genome Atlas (TCGA) database (https://portal.gdc.cancer.gov)The data of SCNA module by the TIMER tool is available in the TIMER database (http://timer.cistrome.org/).

## Abbreviations

BC: Breast cancer
BLBC: Basal-like breast cancer
CI: Confidential interval
CRGs: Cuproptosis-related genes
FC: Fold changes
GDSC: Genomics of drug sensitivity in cancer
GTRP: Genomics of therapeutics response portal
SCNAs: Somatic copy number alterations
TIMER: Tumor immune estimation resource
TME: Tumor microenvironment
TNBC: Triple negative breast cancer
